# 肺癌合并副瘤综合征

**DOI:** 10.3779/j.issn.1009-3419.2014.09.12

**Published:** 2014-09-20

**Authors:** 钢 陈

**Affiliations:** 300052 天津，天津医科大学总医院肺部肿瘤外科 Department of Lung Cancer Surgery, Tianjin Medical University General Hospital, Tianjin 300052, China

肺癌是目前世界上死亡率最高的恶性肿瘤，部分肺癌患者可出现一些少见的症状或体征，表现于胸部以外的脏器而不是肿瘤直接作用或转移引起的，为肺癌的肺外表现。主要包括内分泌系统、神经肌肉系统、结缔组织、血液系统和血管的异常改变，称之为副癌综合征^[1]^。这类症状是由于肺癌细胞产生的某些特殊代谢物引起的，与肺癌所致的局部症状无关。经过肿瘤切除或有效的治疗后这些症状可以消失或减轻，也可以随肿瘤复发而再次出现。肺癌的副癌综合征可以出现于肿瘤诊断之前或同时，部分病例还可能作为首发症状。故早期发现有助于部分肿瘤的早期诊断和治疗，应引起临床医师重视，以防漏诊、误诊。本文就常见肺癌合并的副瘤综合征进行综述。

## 1 内分泌相关

肺癌分泌多种激素及生物活性物质，形成异位激素分泌，有些可引起内分泌功能亢进症状从而造成内分泌相关疾病，例如抗利尿激素、性腺激素等。

### 1.1 库兴综合征

库兴综合征临床表现为低血钾、水肿、高血压、肌无力和体重下降。化验检测血、尿皮质醇含量增高。确诊本病需测定24 h皮质醇含量或小剂量氟美松抑制试验。其发病机理是由于异位分泌的促肾上腺皮质激素(adrenocorticotropic hormone, ACTH)，不断刺激正常肾上腺组织，使其分泌过多肾上腺皮质激素而引起。据统计异位ACTH综合征中肺癌占50%以上^[2]^。治疗须以根除原发肺癌为主要手段，包括手术切除和放化疗方法，兼顾肾上腺皮质激素合成抑制剂和对症治疗。

### 1.2 类癌综合征

类癌综合征临床表现为阵发性皮肤潮红多汗症状，轻中度腹泻，偶见皮炎、痴呆和腹泻三联征^[3]^。常见于小细胞肺癌，早期很难引起患者重视，部分患者伴有哮喘症状。临床化验测血5-HT水平均增高。据统计肺癌引起类癌综合征较胃肠道肿瘤少见。由于肺癌产生的生物活性物质直接进入大循环，故类癌综合征的症状出现的较早，其发生机理与类癌及其转移灶产生过多的5-HT、5-羟色胺酸、缓激肽、组胺等化学介质有关。治疗以手术切除原发病灶为主。

### 1.3 高钙血症

高钙血症可不伴临床症状，测量血钙可高达3.5 mmol/L，但临床检测发现有时并非骨转移，可排除癌细胞直接破骨所致。据统计^[4]^，在没有骨转移的情况下，高钙血症发生率占40%，主要存在于鳞癌和大细胞癌，发生机制与异位甲状旁腺激素与其相关蛋白有关。许多肿瘤都可产生异位甲状旁腺激素，尤以肺癌发生率高。部分高钙血症的癌症患者比正常血钙的癌症患者血浆前列腺素高。肺癌引起的高钙血症需要与原发性甲状旁腺功能亢进鉴别。治疗原则针对原发病为主。其他内分泌综合征抗出现利尿激素分泌异常、促性腺激素分泌异常、高血糖、低血糖等均应引起重视。

### 1.4 男性乳腺增生

肺癌引起的男性患者乳房发育、乳晕增生机制不清，可能与外周血中雌激素水平升高、雄激素水平降低，致使肺癌患者体内性激素失衡和紊乱有关，从而引起男性乳腺增生症状。

## 2 神经肌肉相关

是指由全身性或系统性恶性肿瘤在中枢、周围神经的远隔效应。神经或/及肌肉系统受其影响所引起的一组临床症状群^[5]^。约1%的癌肿患者出现副肿瘤综合征，50%以上患者为肺癌所致。其临床表现多样，最多见的是小细胞肺癌，而神经肌肉症状可早于肿瘤症状，潜伏期可达数周、数月，甚至5年以上^[6]^。

### 2.1 肌无力综合征(Lambert-Eaton综合征)

早期均表现为四肢近端肌肉无力及易疲劳感，下肢重于上肢，逐渐发展到走路不稳、行走不便。肌电图显示有周围神经元性损害。其发生机制可能与自身免疫机制有关。机体对运动神经末梢的膜电位依赖性钙通道产生了自身抗体IgG，引起神经末梢的钙通道减少，当神经冲动到达末梢时，钙离子的流入减少，造成乙酰胆碱的释放减少，终板电位降低，当其降低到神经得以兴奋的域值以下时，神经冲动无法传达到肌肉，不能引起肌肉的效应。副肿瘤综合征患者体内存在有自身抗体，如Anti-Hu、Anti-Yo等抗体，Anti-Hu抗体是一种抗核抗体，存在于细胞核中，可影响脑干、边缘叶、脊髓灰质、脊髓后索及后根、外周感觉神经元。85%以上的患者在肿瘤诊断前即出现了神经症状，直到数月后甚至5年后才找到肿瘤灶。临床表现为多样化及亚急性进展，影像学大多正常。癌性神经病变如发生在原发性肺癌确诊之后并不难获得诊断，如发生在原发肿瘤诊断之前，则需要与其他神经系统疾病进行鉴别。Lambert-Eaton综合征多发生于近端肌群、深腱反射消失或减弱、活动后肌力有所恢复，与重症肌无力相反。Lambert-Eaton综合征对新斯的明不敏感而重症肌无力则疗效明显。Lambert-Eaton综合征肌电图休止时受刺激，动作电位减低，在连续刺激数秒钟之后即升高等亦可与重症肌无力鉴别。肿瘤对神经系统的广泛损害使临床表现复杂多样、只注意神经系统症状而忽略肿瘤对神经系统的损害可能导致误诊，因此对于那些影像学上无相应病灶、无法解释临床表现者，经治疗无效时，一定要想到副肿瘤综合征的可能性，注意复查胸部X线、胸部计算机断层扫描(computed tomography, CT)、脱落细胞检查以及纤维支气管镜检查^[7, 8]^。

### 2.2 多发性肌炎

多发性肌炎为累及横纹肌的特发性炎症性肌病，临床上以对称性近端肌无力为主要表现，病理上以横纹肌肌纤维变性和间质炎症为特点。多发性肌炎为系统性疾病常累及多脏器，肺癌及其他肿瘤和结缔组织病可伴发。40岁以上的多发性肌炎患者合并肿瘤的发生率约为10%-20%^[1]^，肿瘤可于多发性肌炎之前、同时或之后发生。当肌炎呈不典型性、肌电图正常、肌活检不典型或激素抵抗等表现时，需结合年龄、性别及其他临床表现和危险因素除外合并肿瘤之可能。

## 3 骨相关

继发性肥大性骨关节病：临床以杵状指(趾)、广泛性骨膜新骨形成和关节疼痛、积液为主要表现。肥大性骨关节病分原发性和继发性两种。继发性者以肺性肥大性骨关节病最常见，往往先于肺部症状数月或数年出现，多合并于肺癌。其病因学说较多，毒性物质均被认为可能是引起本病的原因。其确诊主要依靠影像学证据，表现为双侧管状骨对称性骨膜增生。增生的骨膜可呈层状、葱皮样或花边样，骨膜新生骨与骨皮质之间多可见一透亮线；关节病变多表现为关节软组织肿胀、关节积液及关节周围骨质疏松等征象。肥大性骨关节病核素骨显像的特征表现为对称性“双轨征”和(或)对称性关节周围放射性浓集。其治疗效果取决于原发病的治疗，原发病缓解后关节症状可完全缓解。对于暂时无法手术或失去手术机会的患者，合理的对症止痛治疗也是十分必要的。Cox-2抑制剂如罗非考昔可有效地缓解疼痛。对于顽固性疼痛可选用双膦酸盐、奥曲肽等药物^[9]^。

## 4 血液相关

肺癌患者同时也可伴发血液相关疾病，但较为少见。

### 4.1 癌性贫血

肺癌患者均在病程某个时期出现不同程度的贫血，晚期肺癌几乎均有贫血，本文所述癌性贫血不包括营养性贫血、失血性贫血、肺癌侵犯骨髓及放、化疗所致的贫血。癌性贫血常发生于肺癌早期，多为轻度贫血，为正细胞正色素血清铁降低，网织红细胞正常，铁剂治疗无效而促红细胞生成素治疗有效，其发生机制是促红细胞生成素不足或骨髓对促红细胞生成素的反应差，骨髓自身的代偿能力减弱等综合引起的。其他血凝系统改变可见白细胞增多症、血小板增多或减少等^[10]^。

### 4.2 癌性血栓

有学者^[11]^对1, 048例肺癌患者分析统计其副癌综合征发生情况，其中104例出现副癌综合征，有8例出现深静脉血栓，占出现副癌综合征例数的7.7%。提示我们出现不明原因的深静脉血栓时应警惕有隐匿性恶性肿瘤的可能。因而对于深静脉血栓患者无论其有无呼吸道症状和体征均应考虑作胸部X线或CT检查，无异常者也应定期随访观察。

## 5 皮肤组织相关

根据以往文献报道，肺癌还可以引起一些皮肤相关性疾病。

### 5.1 红皮病

红皮病又称剥脱性皮炎，是一种以全身皮肤潮红、脱屑为特征的炎症性疾病。此病病因较复杂，某些药物(如青霉素、磺胺类、抗疟药、苯妥英钠、别嘌呤醇和卡马西平等)内用或外用可能致病，某些皮肤病(如特应性皮炎、湿疹、银屑病、毛发红糠疹等)治疗不当或其他刺激加重后也可引起。还可以伴发于原发性皮肤T细胞淋巴瘤、霍奇金病、白血病或其他肿瘤。部分患者常找不到任何原因或诱发原因。红皮病根据典型的临床表现不难诊断，机体原发性疾病也有助于其诊断。治疗上，有明确诱因者应尽早去除，由肿瘤引起者应积极治疗原发疾病有利于改善红皮病预后。

### 5.2 黑棘皮症

黑棘皮症分恶性和良性，恶性黑棘皮症多见于成年人，男性发病率高于女性，可见于肺癌，与肿瘤同时发生占其60%，先有皮肤改变后发生肿瘤者占20%，表现为颜面皮肤灰黑、粗糙、逐渐发展到全身皮肤。

## 6 展望

肺癌的肺外表现复杂，但不能作为诊断的特异性症状，因而，副瘤综合征的临床表现只能作为诊断的一个参考依据。对于肺部肿瘤合并的副瘤综合征，一些症状可能作为首发症状出现，我们应该熟悉了解并及时考虑其相关肿瘤的可能性，这对于肺部肿瘤的早诊早治具有极大的临床意义，动态观察副瘤综合征症状的变化有可能提示其病程的演化及预后。同时作为肺部肿瘤的伴发症状其治疗应着重于原发疾病的治疗，必要时辅以针对合并症的控制措施以期病情顺利恢复。当然，目前对许多副瘤综合征的病因并不清楚，这也需要我们在以后的工作中去总结经验并进一步分析明确其发病机理。
